# Supplementary material for: A Effectiveness-and Efficiency-Based Improved Approach for Measuring Ecological Well-Being Performance in China
Source: Int J Environ Res Public Health. 2023 Jan 22;20(3):2024. doi: 10.3390/ijerph20032024 (PMC9915347; doi:10.3390/ijerph20032024)
Supplement: Supplementary file 1 [file ijerph-20-02024-s001.zip › ijerph-2169727-supplementary.pdf]

Table S1. The value and rank of ECI among 30 provinces from 1997 to 2019 in China

Province	1997(Rank)	1998(Rank)	1999(Rank)	2000(Rank)	2001(Rank)	2002(Rank)	2003(Rank)	2004(Rank)	2005(Rank)	2006(Rank)	2007(Rank)	2008(Rank)	2009(Rank)	2010(Rank)	2011(Rank)	2012(Rank)	2013(Rank)	2014(Rank)	2015(Rank)	2016(Rank)	2017(Rank)	2018(Rank)	2019(Rank)
Beijing	0.2178(4)	0.2043(7)	0.182(5)	0.1617(8)	0.1826(5)	0.1926(5)	0.1959(5)	0.1957(6)	0.18(8)	0.1783(10)	0.1724(9)	0.1629(11)	0.1587(11)	0.1451(14)	0.1361(20)	0.1342(22)	0.1331(22)	0.1337(22)	0.1313(22)	0.1227(22)	0.1257(17)	0.1295(17)	0.1258(20)
Tianjin	0.2211(3)	0.2963(1)	0.1799(6)	0.2043(5)	0.1764(6)	0.167(7)	0.181(7)	0.1795(7)	0.1959(6)	0.1952(7)	0.1901(7)	0.1898(8)	0.1902(8)	0.1906(7)	0.1897(8)	0.1864(8)	0.1759(8)	0.177(8)	0.1707(9)	0.1605(9)	0.1732(7)	0.1675(7)	0.1761(7)
Hebei	0.1297(15)	0.1657(9)	0.1524(9)	0.1382(12)	0.1308(12)	0.129(13)	0.1379(14)	0.1579(9)	0.1598(12)	0.1603(15)	0.1615(12)	0.1561(12)	0.156(13)	0.167(9)	0.1986(7)	0.1957(7)	0.1904(7)	0.1904(7)	0.1794(7)	0.1511(10)	0.1433(12)	0.1378(12)	0.1379(13)
Shanxi	0.1758(7)	0.2882(2)	0.2262(3)	0.2137(3)	0.2167(3)	0.2232(2)	0.2461(2)	0.256(2)	0.2711(3)	0.2674(3)	0.2624(3)	0.2517(3)	0.2392(4)	0.2366(4)	0.2801(4)	0.2795(4)	0.2797(5)	0.2936(5)	0.2879(4)	0.2752(4)	0.2652(4)	0.2707(4)	0.2757(4)
Inner Mongolia	0.1908(6)	0.1872(8)	0.1745(7)	0.1671(6)	0.164(7)	0.1722(6)	0.2381(3)	0.2544(3)	0.3126(1)	0.3127(2)	0.3153(2)	0.3086(2)	0.3131(1)	0.349(1)	0.3792(3)	0.3901(2)	0.3639(3)	0.3847(3)	0.3881(3)	0.3827(2)	0.3779(2)	0.3923(2)	0.4313(2)
Liaoning	0.2138(5)	0.2258(5)	0.2177(4)	0.2137(4)	0.2071(4)	0.1955(4)	0.1959(6)	0.1982(5)	0.2371(4)	0.2465(4)	0.2512(4)	0.2451(4)	0.2432(3)	0.2444(3)	0.2703(5)	0.2692(6)	0.2598(6)	0.2765(6)	0.2779(6)	0.2278(6)	0.2263(6)	0.229(6)	0.2328(6)
Jilin	0.1695(8)	0.1631(10)	0.1599(8)	0.1554(9)	0.1479(8)	0.1442(8)	0.1499(9)	0.1538(11)	0.1693(11)	0.1804(9)	0.1656(11)	0.1638(10)	0.1652(9)	0.165(10)	0.1717(10)	0.1631(11)	0.1618(11)	0.169(11)	0.1676(10)	0.1505(12)	0.1493(10)	0.1474(10)	0.1477(11)
Heilongjiang	0.1466(11)	0.1439(14)	0.1394(12)	0.1415(11)	0.1401(10)	0.1349(11)	0.1424(11)	0.1419(14)	0.1518(15)	0.165(12)	0.1674(10)	0.1652(9)	0.1649(10)	0.1638(11)	0.1739(9)	0.176(9)	0.1682(10)	0.171(10)	0.1669(11)	0.1668(7)	0.1661(8)	0.1674(8)	0.1649(8)
Shanghai	0.2579(1)	0.2649(3)	0.2426(1)	0.2221(2)	0.2192(2)	0.2107(3)	0.2177(4)	0.2299(4)	0.2219(5)	0.2199(5)	0.2139(5)	0.2058(6)	0.1936(7)	0.1803(8)	0.1619(12)	0.157(13)	0.1531(13)	0.1489(16)	0.1433(17)	0.1247(20)	0.1198(21)	0.1304(16)	0.1289(17)
Jiangsu	0.1226(17)	0.139(17)	0.1214(18)	0.1204(15)	0.1193(17)	0.122(16)	0.1301(15)	0.1368(17)	0.1481(17)	0.1632(13)	0.1585(13)	0.1559(13)	0.1565(12)	0.1638(12)	0.1697(11)	0.1691(10)	0.1697(9)	0.1753(9)	0.1742(8)	0.1667(8)	0.1619(9)	0.1579(9)	0.1601(9)
Zhejiang	0.0996(23)	0.1397(16)	0.1328(14)	0.1267(14)	0.119(18)	0.1279(14)	0.1388(13)	0.1456(12)	0.1541(14)	0.1553(16)	0.1461(15)	0.1443(16)	0.1453(15)	0.1424(16)	0.1502(15)	0.1483(14)	0.1515(14)	0.1546(15)	0.1548(14)	0.1256(18)	0.1265(15)	0.1254(19)	0.1268(19)
Anhui	0.0964(24)	0.1055(24)	0.1001(24)	0.0986(26)	0.0918(27)	0.0891(27)	0.0971(27)	0.0974(27)	0.1062(28)	0.1052(27)	0.1061(27)	0.1059(26)	0.0987(28)	0.1031(27)	0.1116(27)	0.1126(26)	0.1122(26)	0.1175(26)	0.1176(26)	0.1233(21)	0.1131(23)	0.1147(24)	0.1239(23)
Fujian	0.0816(29)	0.1016(26)	0.1(25)	0.0951(27)	0.0988(26)	0.0916(26)	0.1024(26)	0.1078(26)	0.1228(25)	0.1288(24)	0.1182(24)	0.1241(23)	0.1269(21)	0.1337(20)	0.1307(22)	0.1432(18)	0.148(16)	0.1437(18)	0.1427(18)	0.1416(13)	0.1291(14)	0.1363(13)	0.1432(12)
Jiangxi	0.0912(26)	0.1041(25)	0.0981(26)	0.105(23)	0.1228(15)	0.1039(22)	0.1172(21)	0.1253(18)	0.13(21)	0.1342(21)	0.1161(25)	0.1107(25)	0.1081(25)	0.1081(26)	0.1192(25)	0.1171(25)	0.1205(25)	0.1224(24)	0.1251(23)	0.1319(15)	0.1237(19)	0.1202(21)	0.124(22)
Shandong	0.1341(13)	0.1456(12)	0.1207(19)	0.1173(17)	0.1134(20)	0.116(18)	0.1262(16)	0.1246(20)	0.1348(18)	0.1409(19)	0.1384(18)	0.1393(17)	0.1397(17)	0.1435(15)	0.1595(13)	0.1591(12)	0.1542(12)	0.164(12)	0.1668(12)	0.1507(11)	0.1464(11)	0.1473(11)	0.1503(10)
Henan	0.0845(28)	0.0938(28)	0.0925(27)	0.0881(28)	0.0808(28)	0.0823(28)	0.0856(28)	0.0928(28)	0.1099(26)	0.1088(26)	0.1068(26)	0.1003(27)	0.0993(27)	0.1002(28)	0.1058(29)	0.1043(29)	0.1043(29)	0.1071(30)	0.1051(30)	0.0804(30)	0.0752(30)	0.0789(30)	0.0833(30)
Hubei	0.1238(16)	0.1337(19)	0.1255(17)	0.1144(19)	0.1168(19)	0.1103(19)	0.1145(22)	0.1213(22)	0.1272(23)	0.1319(23)	0.1226(22)	0.1251(22)	0.1241(22)	0.128(22)	0.1357(21)	0.1366(21)	0.1349(21)	0.1394(21)	0.1374(20)	0.1252(19)	0.1218(20)	0.1232(20)	0.13(16)
Hunan	0.1403(12)	0.1428(15)	0.1361(13)	0.138(13)	0.1405(9)	0.1351(10)	0.1408(12)	0.1404(16)	0.1579(13)	0.1498(17)	0.1364(19)	0.1322(20)	0.1274(20)	0.1238(24)	0.1137(26)	0.1111(27)	0.1076(28)	0.1083(28)	0.1068(29)	0.0981(27)	0.0891(29)	0.0936(28)	0.0973(29)
Guangdong	0.1193(19)	0.1341(18)	0.1315(15)	0.1168(18)	0.1214(16)	0.1203(17)	0.1216(18)	0.1409(15)	0.1483(16)	0.1605(14)	0.1459(16)	0.1459(15)	0.1409(16)	0.1414(17)	0.1449(16)	0.1458(16)	0.1431(18)	0.1452(17)	0.1436(16)	0.1269(17)	0.1254(18)	0.1275(18)	0.1278(18)
Guangxi	0.1219(18)	0.1456(13)	0.1306(16)	0.1667(7)	0.1387(11)	0.1352(9)	0.1499(10)	0.1546(10)	0.1701(9)	0.167(11)	0.1581(14)	0.1505(14)	0.153(14)	0.159(13)	0.1243(24)	0.1262(23)	0.1244(24)	0.1279(23)	0.1227(24)	0.109(25)	0.1109(24)	0.1099(25)	0.1125(25)
Hainan	0.0854(27)	0.0851(29)	0.086(29)	0.0806(29)	0.0604(30)	0.0604(30)	0.0565(30)	0.069(30)	0.0807(30)	0.0851(30)	0.0867(29)	0.0822(30)	0.0853(29)	0.0871(29)	0.0964(30)	0.0991(30)	0.1038(30)	0.1075(29)	0.1071(28)	0.0907(29)	0.0945(28)	0.0903(29)	0.1007(28)
Chongqing	0.111(22)	0.0982(27)	0.1132(21)	0.1011(25)	0.106(22)	0.1052(21)	0.1207(19)	0.1253(19)	0.1334(19)	0.1383(20)	0.1316(21)	0.1329(18)	0.1337(19)	0.1376(18)	0.1394(18)	0.1383(20)	0.1353(20)	0.1404(20)	0.1413(19)	0.0982(26)	0.1004(26)	0.1036(26)	0.1054(27)
Sichuan	0.1118(21)	0.1287(20)	0.0906(28)	0.1123(20)	0.1027(23)	0.1001(25)	0.1068(25)	0.1115(25)	0.1071(27)	0.1052(28)	0.1017(28)	0.0999(28)	0.0996(26)	0.11(25)	0.1092(28)	0.1092(28)	0.1081(27)	0.1151(27)	0.1088(27)	0.0964(28)	0.0979(27)	0.0995(27)	0.1056(26)
Guizhou	0.156(9)	0.2044(6)	0.1482(10)	0.1469(10)	0.1295(13)	0.1222(15)	0.125(17)	0.1244(21)	0.1331(20)	0.1436(18)	0.1436(17)	0.1326(19)	0.1393(18)	0.136(19)	0.1393(19)	0.1407(19)	0.1402(19)	0.1413(19)	0.1359(21)	0.1106(24)	0.1157(22)	0.1191(23)	0.1137(24)
Yunnan	0.0472(30)	0.0747(30)	0.0764(30)	0.0654(30)	0.0618(29)	0.0636(29)	0.0678(29)	0.0714(29)	0.0829(29)	0.091(29)	0.0813(30)	0.0838(29)	0.0849(30)	0.0871(30)	0.1258(23)	0.1213(24)	0.1255(23)	0.1223(25)	0.1181(25)	0.1205(23)	0.1104(25)	0.1195(22)	0.1251(21)
Shaanxi	0.0958(25)	0.1166(23)	0.1124(22)	0.1047(24)	0.1012(25)	0.1027(23)	0.1083(24)	0.1191(23)	0.1276(22)	0.1321(22)	0.1324(20)	0.1308(21)	0.1226(24)	0.1276(23)	0.1436(17)	0.1452(17)	0.1478(17)	0.1597(13)	0.1585(13)	0.1294(16)	0.1355(13)	0.1342(14)	0.1325(14)
Gansu	0.119(20)	0.1175(22)	0.1087(23)	0.1065(22)	0.1024(24)	0.1006(24)	0.1139(23)	0.1143(24)	0.1235(24)	0.1256(25)	0.1201(23)	0.124(24)	0.1231(23)	0.1326(21)	0.1525(14)	0.1483(15)	0.1484(15)	0.1578(14)	0.1528(15)	0.134(14)	0.1262(16)	0.1307(15)	0.1308(15)
Qinghai	0.1478(10)	0.1223(21)	0.1174(20)	0.1123(21)	0.1105(21)	0.1057(20)	0.1185(20)	0.1445(13)	0.1834(7)	0.1954(6)	0.1995(6)	0.2076(5)	0.2068(5)	0.23(5)	0.4194(1)	0.4285(1)	0.4392(1)	0.4574(1)	0.5014(1)	0.481(1)	0.446(1)	0.41(1)	0.4364(1)
Ningxia	0.231(2)	0.2288(4)	0.2318(2)	0.2769(1)	0.2622(1)	0.2451(1)	0.2991(1)	0.2659(1)	0.3116(2)	0.3285(1)	0.3256(1)	0.3139(1)	0.2997(2)	0.3423(2)	0.3964(2)	0.3845(3)	0.3914(2)	0.3977(2)	0.3919(2)	0.3412(3)	0.344(3)	0.3518(3)	0.3624(3)
Xinjiang	0.1324(14)	0.1491(11)	0.1418(11)	0.1204(16)	0.1261(14)	0.1331(12)	0.1523(8)	0.1709(8)	0.1697(10)	0.1874(8)	0.1896(8)	0.1962(7)	0.202(6)	0.211(6)	0.2433(6)	0.2766(5)	0.2986(4)	0.3025(4)	0.2816(5)	0.2576(5)	0.2529(5)	0.2447(5)	0.2498(5)

Table S2. The value and rank of HDI among 30 provinces from 1997 to 2019 in China

Province	1997(Rank)	1998(Rank)	1999(Rank)	2000(Rank)	2001(Rank)	2002(Rank)	2003(Rank)	2004(Rank)	2005(Rank)	2006(Rank)	2007(Rank)	2008(Rank)	2009(Rank)	2010(Rank)	2011(Rank)	2012(Rank)	2013(Rank)	2014(Rank)	2015(Rank)	2016(Rank)	2017(Rank)	2018(Rank)	2019(Rank)
Beijing	0.7852(2)	0.7933(2)	0.7981(2)	0.806(2)	0.8065(2)	0.8107(2)	0.8161(2)	0.8217(2)	0.8271(2)	0.829(2)	0.8389(2)	0.8413(2)	0.8448(3)	0.8642(3)	0.8677(3)	0.8728(3)	0.877(3)	0.8811(3)	0.8839(3)	0.8888(3)	0.8943(3)	0.8975(3)	0.9015(3)
Tianjin	0.7589(3)	0.7627(3)	0.7744(3)	0.7875(3)	0.7885(3)	0.7945(3)	0.8027(3)	0.813(3)	0.8208(3)	0.8279(3)	0.8338(3)	0.8397(3)	0.8457(2)	0.8671(2)	0.8723(2)	0.8778(2)	0.8826(2)	0.8856(2)	0.8895(2)	0.8933(2)	0.8974(2)	0.9018(2)	0.9034(2)
Hebei	0.6912(11)	0.7047(11)	0.713(11)	0.7309(12)	0.7312(12)	0.7398(11)	0.7462(13)	0.7543(12)	0.7596(12)	0.7679(12)	0.7748(12)	0.7843(12)	0.7901(12)	0.7901(19)	0.7957(19)	0.802(20)	0.8089(21)	0.8149(20)	0.8189(20)	0.8244(20)	0.8324(20)	0.837(20)	0.8458(18)
Shanxi	0.6797(14)	0.6871(14)	0.6975(14)	0.7171(14)	0.7205(14)	0.7304(14)	0.7427(14)	0.7534(13)	0.7623(11)	0.773(10)	0.7823(11)	0.7879(11)	0.7922(11)	0.7967(15)	0.8022(15)	0.8094(14)	0.8142(14)	0.8158(18)	0.8183(21)	0.8231(21)	0.8304(21)	0.8333(21)	0.8381(21)
Inner Mongolia	0.658(21)	0.668(21)	0.6733(21)	0.6988(21)	0.6928(21)	0.7035(21)	0.7119(21)	0.7296(18)	0.7382(18)	0.7525(18)	0.7651(18)	0.7737(18)	0.7836(17)	0.8071(11)	0.815(11)	0.8216(11)	0.8253(11)	0.8288(12)	0.8308(12)	0.8373(12)	0.84(13)	0.844(15)	0.8481(15)
Liaoning	0.7326(5)	0.7376(5)	0.7448(5)	0.7558(7)	0.7575(5)	0.7659(5)	0.7713(6)	0.7782(6)	0.7823(7)	0.789(7)	0.7958(8)	0.8013(9)	0.8064(9)	0.8248(8)	0.8285(8)	0.8321(8)	0.8367(8)	0.8393(9)	0.84(9)	0.8407(11)	0.8427(11)	0.8448(13)	0.8475(17)
Jilin	0.7045(10)	0.7105(10)	0.7207(8)	0.7318(11)	0.7357(10)	0.7411(10)	0.7471(10)	0.7528(14)	0.7527(14)	0.7604(14)	0.7686(15)	0.7751(16)	0.7833(18)	0.799(14)	0.804(14)	0.8092(15)	0.8118(17)	0.8127(21)	0.8165(23)	0.8204(23)	0.8207(24)	0.8255(24)	0.8282(25)
Heilongjiang	0.7053(9)	0.7119(9)	0.7152(10)	0.7323(10)	0.7322(11)	0.7394(12)	0.7468(11)	0.7553(11)	0.7578(13)	0.7663(13)	0.7733(13)	0.7796(13)	0.7843(15)	0.8059(12)	0.8106(12)	0.8157(13)	0.8182(13)	0.8195(14)	0.8221(15)	0.822(22)	0.8266(22)	0.8301(23)	0.831(23)
Shanghai	0.808(1)	0.81(1)	0.8161(1)	0.8256(1)	0.8195(1)	0.8222(1)	0.8309(1)	0.8329(1)	0.8396(1)	0.8441(1)	0.8513(1)	0.8547(1)	0.8584(1)	0.8738(1)	0.8784(1)	0.8831(1)	0.8829(1)	0.8881(1)	0.8921(1)	0.8968(1)	0.9033(1)	0.9068(1)	0.9094(1)
Jiangsu	0.7101(7)	0.7165(7)	0.7304(7)	0.7632(6)	0.7518(7)	0.7547(7)	0.7609(7)	0.7715(7)	0.7864(6)	0.7948(6)	0.8044(6)	0.8101(6)	0.8177(6)	0.8366(5)	0.8401(6)	0.8453(6)	0.8527(5)	0.8539(6)	0.8577(6)	0.8613(7)	0.8654(7)	0.8703(7)	0.8765(7)
Zhejiang	0.7197(6)	0.7315(6)	0.74(6)	0.7681(4)	0.7562(6)	0.7636(6)	0.7717(5)	0.7801(5)	0.791(5)	0.8031(5)	0.8107(5)	0.8183(5)	0.8269(5)	0.8351(6)	0.8402(5)	0.8483(5)	0.8526(6)	0.8563(5)	0.8614(5)	0.8661(5)	0.8733(5)	0.8795(5)	0.8843(5)
Anhui	0.6485(23)	0.6553(24)	0.664(23)	0.6976(22)	0.6886(22)	0.6941(23)	0.7118(22)	0.7172(23)	0.7146(25)	0.7316(25)	0.7393(26)	0.7533(25)	0.7642(25)	0.7759(25)	0.7885(23)	0.7953(23)	0.8045(23)	0.8115(23)	0.8198(18)	0.8255(19)	0.8326(19)	0.839(19)	0.8449(19)
Fujian	0.7076(8)	0.7121(8)	0.719(9)	0.7519(8)	0.7435(8)	0.7472(9)	0.755(8)	0.7573(10)	0.7706(8)	0.7835(9)	0.7919(9)	0.8015(8)	0.8138(7)	0.8296(7)	0.8328(7)	0.8398(7)	0.8454(7)	0.8512(7)	0.855(7)	0.8634(6)	0.8719(6)	0.8754(6)	0.8811(6)
Jiangxi	0.6487(22)	0.6537(25)	0.6615(25)	0.6891(25)	0.6859(25)	0.6924(24)	0.708(24)	0.7163(24)	0.7223(23)	0.7364(23)	0.7507(22)	0.7618(23)	0.775(21)	0.7849(21)	0.7952(20)	0.8031(19)	0.8118(18)	0.8176(16)	0.8217(17)	0.8284(16)	0.837(15)	0.845(12)	0.8542(11)
Shandong	0.6912(12)	0.6993(12)	0.7106(12)	0.746(9)	0.7406(9)	0.751(8)	0.751(9)	0.7625(8)	0.7705(9)	0.7865(8)	0.7961(7)	0.8028(7)	0.8104(8)	0.8136(9)	0.8184(9)	0.8253(9)	0.8335(9)	0.8397(8)	0.8425(8)	0.8503(8)	0.8591(8)	0.8618(8)	0.865(8)
Henan	0.6671(18)	0.6759(18)	0.6787(20)	0.7122(16)	0.711(16)	0.7192(16)	0.726(17)	0.7385(15)	0.7443(15)	0.7564(15)	0.7679(16)	0.7771(15)	0.7863(14)	0.7929(17)	0.8001(17)	0.8073(16)	0.8118(19)	0.819(15)	0.8233(14)	0.8289(14)	0.8372(14)	0.8437(16)	0.8492(14)
Hubei	0.6717(16)	0.6789(17)	0.6852(18)	0.7114(17)	0.7101(17)	0.706(20)	0.7196(18)	0.7267(21)	0.7316(21)	0.7446(21)	0.7554(21)	0.7648(22)	0.7717(23)	0.795(16)	0.8011(16)	0.8051(17)	0.8125(16)	0.8168(17)	0.8221(16)	0.8286(15)	0.8344(18)	0.8427(17)	0.8477(16)
Hunan	0.6737(15)	0.6808(16)	0.686(17)	0.7074(19)	0.7063(18)	0.7126(18)	0.7189(19)	0.7288(19)	0.7338(20)	0.7461(20)	0.7583(20)	0.7656(21)	0.7766(20)	0.7904(18)	0.7962(18)	0.8039(18)	0.8132(15)	0.8198(13)	0.8265(13)	0.8331(13)	0.8403(12)	0.8463(11)	0.8517(12)
Guangdong	0.7341(4)	0.7432(4)	0.7508(4)	0.7678(5)	0.7647(4)	0.7761(4)	0.7852(4)	0.7963(4)	0.8082(4)	0.8194(4)	0.83(4)	0.8361(4)	0.8428(4)	0.8405(4)	0.8441(4)	0.85(4)	0.8551(4)	0.8596(4)	0.8657(4)	0.8715(4)	0.879(4)	0.8846(4)	0.8891(4)
Guangxi	0.6584(20)	0.6743(20)	0.6831(19)	0.7103(18)	0.7059(20)	0.7105(19)	0.7188(20)	0.7285(20)	0.7363(19)	0.7514(19)	0.7609(19)	0.7691(19)	0.7788(19)	0.784(22)	0.7924(22)	0.8008(21)	0.8091(20)	0.8156(19)	0.8196(19)	0.8284(17)	0.8368(16)	0.8441(14)	0.8506(13)
Hainan	0.686(13)	0.6941(13)	0.7018(13)	0.7276(13)	0.7231(13)	0.7386(13)	0.7468(12)	0.7598(9)	0.7635(10)	0.7729(11)	0.7856(10)	0.7924(10)	0.8023(10)	0.8016(13)	0.8105(13)	0.8181(12)	0.8243(12)	0.8319(11)	0.836(11)	0.8445(10)	0.8522(10)	0.859(10)	0.8639(10)
Chongqing	0.6715(17)	0.6819(15)	0.69(15)	0.7157(15)	0.713(15)	0.7234(15)	0.7363(15)	0.7356(16)	0.7439(16)	0.7564(16)	0.7694(14)	0.7779(14)	0.787(13)	0.8085(10)	0.8162(10)	0.8222(10)	0.8298(10)	0.8348(10)	0.8394(10)	0.8497(9)	0.8577(9)	0.8594(9)	0.8645(9)
Sichuan	0.6473(25)	0.6612(22)	0.6656(22)	0.6966(24)	0.688(24)	0.6967(22)	0.7073(25)	0.716(25)	0.7104(26)	0.7292(26)	0.7425(25)	0.7493(26)	0.7595(26)	0.7817(23)	0.7884(24)	0.7953(24)	0.8003(25)	0.8037(25)	0.8056(25)	0.8099(25)	0.8182(25)	0.8222(25)	0.8283(24)
Guizhou	0.5676(29)	0.5689(29)	0.5883(30)	0.6225(30)	0.6147(30)	0.6276(30)	0.634(30)	0.6496(30)	0.6524(30)	0.6689(30)	0.6856(30)	0.7003(30)	0.7133(30)	0.7252(30)	0.7326(30)	0.7412(30)	0.7527(30)	0.7582(30)	0.7604(29)	0.7703(30)	0.782(30)	0.7894(30)	0.7945(30)
Yunnan	0.6011(27)	0.6084(27)	0.6186(27)	0.6564(27)	0.6422(27)	0.6432(29)	0.6545(29)	0.6756(27)	0.6734(29)	0.6908(28)	0.7001(29)	0.7147(28)	0.7216(29)	0.7372(28)	0.7429(29)	0.751(28)	0.7578(28)	0.7642(28)	0.7671(28)	0.7753(28)	0.7834(29)	0.7909(29)	0.7986(28)
Shaanxi	0.648(24)	0.6592(23)	0.6633(24)	0.6972(23)	0.6883(23)	0.692(25)	0.7081(23)	0.7199(22)	0.7274(22)	0.7373(22)	0.7464(24)	0.756(24)	0.7651(24)	0.7884(20)	0.7933(21)	0.8004(22)	0.8067(22)	0.8082(24)	0.8149(24)	0.8189(24)	0.8235(23)	0.8303(22)	0.8374(22)
Gansu	0.5962(28)	0.6004(28)	0.6161(28)	0.6522(28)	0.6421(28)	0.6539(27)	0.6645(27)	0.675(28)	0.6796(27)	0.6847(29)	0.7003(28)	0.7112(29)	0.7227(28)	0.7451(27)	0.7547(27)	0.7643(27)	0.7735(27)	0.7761(27)	0.7746(27)	0.7871(27)	0.7896(28)	0.7911(28)	0.7948(29)
Qinghai	0.5505(30)	0.5608(30)	0.6034(29)	0.6437(29)	0.6242(29)	0.6467(28)	0.6592(28)	0.672(29)	0.6753(28)	0.6971(27)	0.7088(27)	0.7222(27)	0.7352(27)	0.7337(29)	0.7479(28)	0.7508(29)	0.7546(29)	0.7625(29)	0.7595(30)	0.7746(29)	0.7913(27)	0.7968(27)	0.8006(27)
Ningxia	0.6266(26)	0.6356(26)	0.65(26)	0.6831(26)	0.6797(26)	0.6913(26)	0.7006(26)	0.7148(26)	0.7161(24)	0.734(24)	0.7472(23)	0.7658(20)	0.7749(22)	0.7659(26)	0.7728(26)	0.7822(26)	0.7882(26)	0.7935(26)	0.7966(26)	0.809(26)	0.8141(26)	0.8146(26)	0.8221(26)
Xinjiang	0.6667(19)	0.6759(19)	0.6884(16)	0.7042(20)	0.7063(19)	0.7143(17)	0.7266(16)	0.7353(17)	0.7404(17)	0.7526(17)	0.7673(17)	0.7745(17)	0.7843(16)	0.7783(24)	0.7859(25)	0.7948(25)	0.8018(24)	0.8124(22)	0.8172(22)	0.8265(18)	0.836(17)	0.8399(18)	0.8436(20)

Table S3. The efficiency value and rank of EWP among 30 provinces from 1997 to 2019 in China

Province	1997(Rank)	1998(Rank)	1999(Rank)	2000(Rank)	2001(Rank)	2002(Rank)	2003(Rank)	2004(Rank)	2005(Rank)	2006(Rank)	2007(Rank)	2008(Rank)	2009(Rank)	2010(Rank)	2011(Rank)	2012(Rank)	2013(Rank)	2014(Rank)	2015(Rank)	2016(Rank)	2017(Rank)	2018(Rank)	2019(Rank)
Beijing	0.7332(20)	0.7418(16)	0.9315(16)	1.066(4)	0.8928(18)	1.0322(9)	0.8932(17)	0.8683(19)	1.0515(1)	1.0273(2)	1.0471(2)	0.9988(12)	0.9505(14)	1.0385(5)	0.9963(11)	1.0238(5)	1.0271(7)	0.9591(11)	1.0414(2)	1.2142(1)	1.1214(2)	1.1391(1)	1.1466(1)
Tianjin	0.7564(19)	0.7531(15)	1.032(6)	1.0226(14)	0.9531(17)	1.0295(12)	0.9346(13)	1.0351(5)	0.9865(10)	0.9284(14)	0.9729(14)	0.9779(13)	0.9293(16)	0.9767(13)	0.9476(14)	1.0214(6)	1.0402(3)	1.0265(1)	1.0487(1)	1.0843(4)	0.9835(15)	1.0615(3)	1.0455(4)
Hebei	0.5811(21)	0.551(22)	0.5975(23)	0.689(23)	0.7253(23)	0.7425(23)	0.6708(23)	0.6718(23)	0.7309(23)	0.7051(21)	0.66(22)	0.6771(23)	0.6487(24)	0.6608(24)	0.8537(17)	0.9329(16)	1.0082(13)	1.019(2)	0.72(17)	1.0077(15)	1.0099(9)	1.0007(12)	0.6402(20)
Shanxi	0.5211(24)	0.4068(29)	0.479(26)	0.5188(26)	0.6858(24)	1.0093(15)	1.0038(12)	0.7545(21)	0.5779(24)	0.5858(23)	0.591(23)	0.8094(20)	0.77(21)	0.9029(17)	0.7843(20)	0.9137(18)	0.941(17)	0.9353(13)	1.0079(11)	1.0081(12)	0.8646(16)	0.6126(21)	1.0177(7)
Inner Mongolia	0.4006(28)	0.4489(27)	0.474(27)	0.5052(27)	0.4775(26)	0.4671(27)	0.4209(27)	0.4716(28)	0.4628(28)	0.4925(25)	0.5027(26)	0.93(15)	1.0032(12)	0.734(21)	0.8095(19)	1.0077(10)	0.698(23)	0.5132(26)	0.4676(26)	0.4551(26)	0.4439(26)	0.4667(25)	0.4566(26)
Liaoning	0.4248(26)	0.4292(28)	0.4296(29)	0.4274(28)	0.421(28)	0.4358(28)	0.4227(26)	0.4825(27)	0.4726(27)	0.4788(27)	0.4652(29)	0.4559(29)	0.4534(29)	0.462(28)	0.4533(28)	0.4514(27)	0.4744(27)	0.421(27)	0.391(27)	0.4091(27)	0.3962(27)	0.4068(27)	0.4024(27)
Jilin	0.4247(27)	0.4569(26)	0.4377(28)	0.4243(29)	0.4029(29)	0.4075(29)	0.3995(29)	0.41(29)	0.4121(29)	0.4047(29)	0.5729(25)	0.5566(25)	0.569(25)	0.5715(25)	0.5677(25)	0.5992(25)	0.6054(25)	0.5463(25)	0.5011(25)	0.518(24)	0.5074(24)	0.5228(24)	0.5366(24)
Heilongjiang	0.5258(23)	0.5212(24)	0.523(25)	0.5386(25)	0.4578(27)	0.4703(26)	0.4174(28)	0.6808(22)	0.8476(21)	0.4882(26)	0.4964(27)	0.498(27)	0.5161(27)	0.5482(26)	0.5588(26)	0.5794(26)	0.604(26)	0.5666(24)	0.5138(24)	0.4697(25)	0.462(25)	0.4576(26)	0.4594(25)
Shanghai	1.0113(14)	0.6907(18)	0.8441(20)	0.9265(22)	0.865(20)	1.0594(3)	1.0277(3)	0.8923(18)	0.9012(17)	0.8838(19)	0.9278(15)	0.8791(19)	0.8473(19)	0.874(19)	0.9478(13)	0.9643(14)	1.043(1)	0.8876(15)	0.8637(13)	1.0739(5)	1.1297(1)	0.9886(13)	1.1201(2)
Jiangsu	1.0071(17)	0.7355(17)	0.9424(15)	1.0531(7)	1.0067(14)	1.0076(16)	1.0257(5)	1.0052(12)	0.9336(15)	0.8577(20)	0.877(20)	0.8961(18)	0.9345(15)	0.9408(14)	0.8521(18)	0.8419(20)	0.8614(19)	0.7231(19)	0.6837(18)	0.6719(20)	0.6726(20)	0.9762(14)	1.0057(11)
Zhejiang	1.0573(6)	0.8697(9)	1.017(9)	1.0346(12)	1.0162(10)	1.0068(18)	0.9069(15)	0.9555(15)	0.8713(20)	0.8854(18)	0.9103(18)	0.9409(14)	0.8593(18)	0.8788(18)	0.7734(21)	0.8059(21)	0.7819(20)	0.7178(20)	0.6727(19)	0.7557(18)	0.7462(18)	0.7785(17)	0.783(17)
Anhui	1.0098(16)	0.8137(12)	1.006(14)	1.0457(9)	1.0086(13)	1.0194(14)	1.0153(9)	1.0203(7)	0.9374(14)	1.0167(7)	1.0191(6)	1.0183(5)	1.03(2)	1.0315(8)	1.0141(7)	1.018(7)	1.0155(12)	1.0079(8)	1.0097(10)	1.0043(16)	1.0011(14)	1.004(11)	0.6572(19)
Fujian	1.2865(2)	1.0462(1)	1.0257(7)	1.1105(1)	1.0246(7)	1.0746(1)	1.0289(2)	1.0289(6)	1.0082(9)	1.0067(11)	1.0276(5)	1.0023(11)	1.0175(9)	1.0196(10)	1.0213(4)	0.9699(13)	0.9238(18)	0.857(17)	0.7993(16)	1.0131(11)	1.0028(13)	0.9403(15)	1.0071(9)
Jiangxi	1.0669(4)	1.0076(5)	1.0395(5)	1.0112(20)	1.0447(3)	1.0311(11)	0.8685(21)	0.9326(16)	0.973(11)	0.9794(13)	1.0165(7)	1.0166(7)	1.0226(7)	1.0338(6)	0.9923(12)	1.0261(4)	1.0229(9)	1.014(3)	1.0279(4)	1.008(14)	1.0046(12)	1.0051(9)	0.9422(14)
Shandong	0.7794(18)	0.787(14)	0.9074(18)	0.9428(21)	0.9576(16)	0.9577(20)	0.888(19)	1.0107(11)	0.9688(12)	0.9009(17)	0.9228(16)	0.903(17)	0.9074(17)	0.937(15)	0.8701(16)	0.8924(19)	1.0168(11)	0.8874(16)	0.6265(21)	0.62(21)	0.6228(21)	0.6359(19)	0.6373(21)
Henan	1.0248(12)	1.0177(4)	1.0157(11)	1.0354(11)	1.02(9)	1.032(10)	1.0205(7)	1.043(3)	1.0125(7)	1.0175(6)	1.0062(12)	1.0329(3)	1.0298(3)	1.0521(4)	1.0212(5)	1.0528(1)	1.031(6)	1.0121(6)	1.0264(6)	1.103(3)	1.115(3)	1.0425(4)	1.064(3)
Hubei	0.5554(22)	0.5466(23)	0.5658(24)	0.5785(24)	0.5232(25)	0.537(25)	0.5324(24)	0.5303(25)	0.5603(25)	0.6156(22)	0.6902(21)	0.6666(24)	0.6749(23)	0.6942(22)	0.6882(23)	0.6963(23)	0.7191(21)	0.6719(21)	0.6417(20)	0.6048(22)	0.5972(23)	0.6228(20)	0.6083(22)
Hunan	1.032(10)	0.8385(11)	1.0431(3)	1.0211(15)	0.8707(19)	0.9431(21)	0.8926(18)	0.9119(17)	0.7865(22)	0.9263(15)	1.0085(11)	1.0038(10)	1.0096(11)	1.0165(11)	1.007(9)	1.0038(12)	1.0243(8)	1.0129(4)	1.0371(3)	1.0471(7)	1.0333(5)	1.0187(6)	1.0063(10)
Guangdong	1.0311(11)	0.8017(13)	0.8732(19)	1.0606(5)	1.0148(11)	1.0214(13)	1.0254(6)	1.017(9)	0.9616(13)	1.0247(4)	1.0164(8)	0.9095(16)	0.9589(13)	1.027(9)	0.9265(15)	0.9313(17)	0.9771(14)	0.9082(14)	1.0191(8)	1.0142(10)	1.0168(7)	1.0067(8)	1.0108(8)
Guangxi	1.0606(5)	1.0075(6)	1.047(2)	1.03(13)	1.0243(8)	1.0381(6)	0.8991(16)	0.9576(14)	1.0393(2)	1.0149(9)	1.0151(9)	1.0174(6)	1.0264(5)	1.0323(7)	1.0215(3)	1.006(11)	1.0426(2)	0.9705(10)	1.0212(7)	1.0439(8)	1.0069(10)	1.0044(10)	0.8352(16)
Hainan	1.0433(8)	1.0253(2)	1.0479(1)	1.0602(6)	1.1298(1)	1.0438(4)	1.0934(1)	1.0495(1)	1.0378(3)	1.1079(1)	1.0426(4)	1.0469(2)	1.0589(1)	1.1066(1)	1.0414(1)	1.0426(2)	1.0372(5)	1.0125(5)	1.0275(5)	1.1477(2)	1.0458(4)	1.0819(2)	1.0286(5)
Chongqing	1.0421(9)	1.021(3)	1.0239(8)	1.0928(2)	1.032(6)	1.0323(8)	1.0107(11)	1.0173(8)	1.0096(8)	0.9127(16)	1.0621(1)	1.0124(8)	1.0146(10)	1.0141(12)	1.0119(8)	1.0129(9)	1.0177(10)	1.0053(9)	0.8316(14)	1.051(6)	1.0289(6)	1.0316(5)	1.0248(6)
Sichuan	1.0572(7)	0.6853(19)	1.017(10)	1.0144(18)	1.0045(15)	0.8645(22)	0.7325(22)	0.7558(20)	0.8781(19)	1.008(10)	0.9849(13)	1.0085(9)	1.0266(4)	0.906(16)	1.0041(10)	1.0174(8)	1.0387(4)	0.8068(18)	0.8215(15)	0.7291(19)	0.6902(19)	0.6961(18)	0.6661(18)
Guizhou	1.0752(3)	0.6525(20)	1.0426(4)	1.0408(10)	1.0614(2)	1.0384(5)	1.013(10)	1.0356(4)	1.0343(4)	1.0187(5)	1.0119(10)	1.0613(1)	1.0221(8)	1.0592(3)	1.0283(2)	0.9357(15)	0.9625(15)	0.9489(12)	1.0164(9)	1.0403(9)	0.8342(17)	0.8935(16)	1.0051(12)
Yunnan	1.3814(1)	0.9575(7)	1.0123(12)	1.0702(3)	1.0382(4)	1.0648(2)	1.0193(8)	1.0488(2)	1.0216(5)	1.0257(3)	1.0445(3)	1.0229(4)	1.0252(6)	1.0736(2)	1.0149(6)	1.0329(3)	0.9592(16)	1.0117(7)	0.9277(12)	1.0014(17)	1.016(8)	1.0107(7)	0.9159(15)
Shaanxi	1.0101(15)	0.8572(10)	0.9308(17)	1.053(8)	1.0137(12)	1.0071(17)	1.0269(4)	1.0126(10)	1.0198(6)	1.015(8)	0.9021(19)	0.7892(22)	0.8262(20)	0.791(20)	0.7392(22)	0.7161(22)	0.7011(22)	0.6187(22)	0.5609(22)	0.5983(23)	0.6034(22)	0.5751(23)	0.5979(23)
Gansu	0.4803(25)	0.6283(21)	0.7785(21)	1.0175(16)	1.0361(5)	1.0352(7)	0.8794(20)	0.9738(13)	0.8878(18)	1.0041(12)	0.9192(17)	0.8083(21)	0.7159(22)	0.6866(23)	0.6208(24)	0.642(24)	0.6348(24)	0.5803(23)	0.5568(23)	1.0081(13)	1.0065(11)	0.5885(22)	1.0008(13)
Qinghai	0.388(29)	0.5147(25)	0.6537(22)	1.0123(19)	0.7594(22)	1.0052(19)	0.9292(14)	0.5733(24)	0.4732(26)	0.4702(28)	0.4844(28)	0.4923(28)	0.4829(28)	0.4597(29)	0.4132(29)	0.4355(28)	0.4016(29)	0.3718(29)	0.3306(29)	0.3649(29)	0.3916(28)	0.3667(29)	0.3671(29)
Ningxia	0.3287(30)	0.3612(30)	0.3609(30)	0.3404(30)	0.3194(30)	0.3361(30)	0.3101(30)	0.3549(30)	0.3476(30)	0.3531(30)	0.3758(30)	0.3835(30)	0.4113(30)	0.346(30)	0.3089(30)	0.3239(30)	0.326(30)	0.3046(30)	0.2871(30)	0.3089(30)	0.3116(30)	0.3211(30)	0.3219(30)
Xinjiang	1.0208(13)	0.9042(8)	1.0077(13)	1.0146(17)	0.8448(21)	0.5488(24)	0.4631(25)	0.5012(26)	0.9041(16)	0.5557(24)	0.5871(24)	0.5466(26)	0.5298(26)	0.5088(27)	0.456(27)	0.4205(29)	0.4054(28)	0.3934(28)	0.3842(28)	0.3694(28)	0.3778(29)	0.3846(28)	0.3945(28)
